# Parent and Peer Attachments in Adolescence and Paternal Postpartum Mental Health: Findings From the ATP Generation 3 Study

**DOI:** 10.3389/fpsyg.2021.672174

**Published:** 2021-05-28

**Authors:** Jacqui A. Macdonald, Christopher J. Greenwood, Primrose Letcher, Elizabeth A. Spry, Kayla Mansour, Jennifer E. McIntosh, Kimberly C. Thomson, Camille Deane, Ebony J. Biden, Ben Edwards, Delyse Hutchinson, Joyce Cleary, John W. Toumbourou, Ann V. Sanson, Craig A. Olsson

**Affiliations:** ^1^Faculty of Health, School of Psychology, Centre for Social and Early Emotional Development, Deakin University, Geelong, VIC, Australia; ^2^Centre for Adolescent Health, Murdoch Children's Research Institute, Parkville, VIC, Australia; ^3^Department of Paediatrics, Royal Children's Hospital, University of Melbourne, Parkville, VIC, Australia; ^4^The Bouverie Centre, School of Psychology & Public Health, La Trobe University, Melbourne, VIC, Australia; ^5^Human Early Learning Partnership, School of Population and Public Health, University of British Columbia, Vancouver, BC, Canada; ^6^Centre for Health Evaluation and Outcome Sciences, Providence Health Care Research Institute, Vancouver, BC, Canada; ^7^Centre for Social Research and Methods, Australian National University, Canberra, ACT, Australia; ^8^National Drug and Alcohol Research Centre, University of New South Wales, Sydney, NSW, Australia

**Keywords:** father, mental health, parents, peers, relationship, postpartum, longitudinal, cohort studies

## Abstract

**Background:** When adolescent boys experience close, secure relationships with their parents and peers, the implications are potentially far reaching, including lower levels of mental health problems in adolescence and young adulthood. Here we use rare prospective intergenerational data to extend our understanding of the impact of adolescent attachments on subsequent postpartum mental health problems in early fatherhood.

**Methods:** At age 17–18 years, we used an abbreviated Inventory of Parent and Peer Attachment to assess trust, communication, and alienation reported by 270 male participants in their relationships with mothers, fathers, and peers. More than a decade later, we assessed the adult males, now fathers, at 12 months postpartum (*N* = 409 infant offspring) for symptoms of depression, anxiety, and stress. Logistic regression was used to examine the extent to which attachment dimensions predicted paternal postpartum mental health, adjusting for potential confounding, and with assessment for interactions between parent and peer attachments.

**Results:** Trust in mothers and peers, and good communication with fathers during adolescence, were associated with 5 to 7 percentage point reductions in postpartum mental health symptoms in early fatherhood. Weak evidence of parent-peer interactions suggested secure attachments with either parent or peer may compensate for an insecure attachment with the other.

**Conclusions:** Our results suggest that fostering trust and communication in relationships that adolescent boys have with parents and peers may have substantial effects on rates of paternal postpartum mental health problems. The protective benefits may be preventative in intergenerational cycles of risk for mental health problems.

## Introduction

Symptoms of common mental health problems (depression and anxiety) are reported by ~10 per cent of fathers of infant children (Giallo et al., 2013; Cameron et al., 2016; Leach et al., 2016; Glasser and Lerner-Geva, 2019). Public health ramifications extend to partners, usually mothers, with reduced levels of postnatal support (Pilkington et al., 2015), and heightened probability of their own mental health problems (Paulson et al., 2010, 2016). Intergenerationally, children of depressed or anxious fathers are also at greater risk of behavioural, social, and emotional problems (Gentile and Fusco, 2017). Compared to mothers, fewer health services screen and treat paternal postpartum mental health problems and considerable barriers exist to paternal support at levels of both the individual (e.g., masculine stoicism; Mansfield et al., 2003; Oliffe and Phillips, 2008) and the health system (e.g., maternal-centric culture; Panter-Brick et al., 2014). In this context, there is a critical need to identify targets for prevention of postpartum mental health problems in fathers. Understanding factors in the developmental history of postpartum mental health problems can also guide treatment options and improve outcomes (Goodman and Dimidjian, 2012).

Emerging evidence from prospective longitudinal cohort studies points to the origins of paternal mental health problems typically existing prior to offspring conception (Thomson et al., 2020). In one sample of expectant fathers with partners in the third trimester of pregnancy (*N* = 295 pregnancies to 214 men), 68% prospectively reported a preconception history of common mental health disorders (Spry et al., 2018). Further, Thomson et al. (2020) reported that 83% of fathers who exhibited depressive symptoms at 12 months postpartum had a history of preconception mental health problems in adolescence and young adulthood. These studies indicate pathways of homotypic continuity in men's symptoms of mental health problems and suggest that prevention efforts should be focused on risk mitigation prior to offspring conception.

However, there is a lack of prospective research examining the preconception contexts associated with paternal postpartum mental health problems. Given that histories of mental illness are not always apparent in those with postpartum mental health problems (Patton et al., 2015; Thomson et al., 2020), and where they do exist, that a prior history does not fully account for the variance in postpartum symptoms, there is a need to extend investigations into other formative developmental domains. One of the most aetiologically relevant of these is the interpersonal domain. In particular, attachment theory, and evidence from retrospective studies, suggest that secure relationships with parents and peers prior to adulthood forge protective foundations that buffer against postpartum mental health risk (Allen and Miga, 2010). Conversely, insecure preconception parent and peer relationships are likely implicated in subsequent postpartum psychological risk (Lee and Hankin, 2009; Mikulincer and Shaver, 2012; McDougall and Vaillancourt, 2015).

In adolescence and young adulthood, the measurement of parent and peer attachment relationships commonly include the dimensions of mutual trust (i.e., mutual understanding in the relationship), quality of communication (i.e., perceptions that others are sensitive and responsive to emotional needs and disclosures), and degree of anger and alienation (i.e., a sense of isolation and detachment) (Gorrese and Ruggieri, 2012). These are captured in the Inventory of Parent and Peer Attachment (IPPA; Armsden and Greenberg, 1987), a self-report measure of the young person's relationships with their mother, father, and peers. The dimensions align to core features of ‘internal working models’ of attachment that indicate the availability of others in times of need. The evidence from cross-sectional and longitudinal studies with outcomes in adolescence or young adulthood (i.e., prior to next-generation conception), is that trust, communication, and alienation with parents and peers are associated with depression and anxiety (Agerup et al., 2015; Gorrese, 2016). Longitudinal studies with preconception assessments are yet to examine whether these associations extend into the postnatal period when there is potential for intergenerational effects on family functioning and child development. Moreover, this line of enquiry is yet to cast a lens on fathers.

The lack of research on fathers is not surprising given a dominant focus on developmental pathways to parenthood in women, for whom parental caregiving roles are typically socialised in deeply embedded, gender-based interactions (Wood and Eagly, 2012; Eagly and Wood, 2016). In general, there is considerably less developmental preparation of males for fatherhood (Baldwin et al., 2018). Men without children often report being uncertain about what future fatherhood is likely to entail in terms of both pragmatic functions and emotional adjustment (Kings et al., 2017). Men with children often report having entered fatherhood lacking critical insights into their partner's and children's needs and into the extent of psychological upheaval that the new role would entail (Darwin et al., 2017; Baldwin et al., 2018). They also report limited acknowledgement and a lack of support from clinical and community support services (Baldwin et al., 2018, 2019) often being told that, in the postpartum period, their needs are comparatively unimportant (Baldwin et al., 2018; Pfitzner et al., 2018). This results in men's minimisation of symptoms and diversion of attention away from their own psychological vulnerability (Darwin et al., 2017; Burgess and Goldman, 2018). The vastly different gender-based, developmental and postpartum experiences related to preparation for and engagement in parenting may manifest in distinct pathways of risk for postpartum mental health vulnerability.

In one retrospective study with assessments taken postpartum, psychologically distressed fathers, compared to non-distressed fathers, reported lower care and higher overprotection (psychological control) from their own fathers and mothers up to age 16 years (Boyce et al., 2007). Retrospective postpartum assessments may be subject to recollection biases and may be particularly influenced by current psychological distress (Bryant et al., 1989; Amato, 1991; Pless and Pless, 1995). To our knowledge there is no prospective evidence of these relationships as assessed with the IPPA, although multi-generational, longitudinal studies do provide evidence that related constructs such as neglect and harsh parenting experienced during childhood and adolescence are linked to subsequent risk for mental health problems when parenting the next generation (Greene et al., 2020; Neppl et al., 2020).

Also lacking are prospective studies of how preconception affiliative relations (i.e., friendships with peers) may increase risk or protection for mental health during the postpartum period. Positive relationships with peers prior to transitioning into parenthood indicate normative psychological adjustment (Laible et al., 2000, 2004; Laible, 2007). Wilkinson (2004) contends that peer attachments inform the evaluation of the self and the construction of identity, which when deficient heighten risk for mental health problems. In new parents, a history of close and available relationships with peers may signal positive identity development and the availability of a broader protective network for support during major life events and transitions such as early parenthood (Bäckström et al., 2020).

It is also possible that the qualities of parent and peer relationships may interact to influence subsequent mental health and well-being. One hypothesis suggests that peer relationships represent an extension of family attachments whereby internal working models of parent availability and sensitivity inform representations of others, including peers (Bowlby, 1969). Gorrese and Ruggieri (2012) found supporting evidence for this but noted patterns of trust, communication and alienation in peer relationships were more similar to mother than father relationships. They argued this was potentially because mothers were more likely to be the primary attachment figure (Gorrese and Ruggieri, 2012). An alternative model suggests that qualities of either peer or parent relationships in adolescence may compensate for unmet needs (Wilkinson, 2004) or fulfil new developmental needs, particularly approaching the transition to adulthood (Buist et al., 2002; Arnett, 2004). How parent and peer relationships interact may inform subsequent risk for mental health problems across the transition to fatherhood; however, this has yet to be investigated. The qualities of each may compound or create a buffer against the influence of the other on future psychological risk.

The aim of this paper is to gain insights into preconception predictors of risk for paternal postpartum mental health problems that may represent prevention targets or inform treatment approaches. We draw on rare longitudinal data of males spanning late adolescence to 1 year after the birth of their offspring. We assess communication, trust and alienation in relationships with mothers, fathers and peers (at 17–18 years) and examine associations with mental health symptoms of depression, anxiety, and stress that are assessed more than a decade later, at 1 year postpartum. We further examine the inter-relationships between parent and peer histories on postpartum mental health to understand if a deficit in one might be counteracted by the protective nature of the other or whether there are compounding effects of poor relationships with both.

## Methods

### Participants

Participants were from the Australian Temperament Project (ATP), a 16-wave longitudinal study tracking the psychosocial development of young people from infancy to adulthood. The baseline sample consisted of 2,443 infants aged between 4 and 8 months (Generation 2; G2) and their parents (Generation 1; G1), recruited in 1983 from urban and rural areas and representative of the state of Victoria, Australia. Subsequently, families have been invited to participate via mail surveys approximately every 2 years until 19–20 years and every 4 years thereafter (Vassallo and Sanson, 2013).

The ongoing, prospective ATP Generation 3 (G3) study commenced when G2 participants were aged 29–35 years, with recruitment of G3 infant offspring. Identification of pregnancies occurred via participant email or phone every 6 months between 2012 and 2018. Telephone or web-based surveys were conducted with parents at the third trimester of pregnancy, and at 2 months and 1 year postpartum. The present study used data collected from G2 cohort participants at the 1 year postpartum interview for each of their G3 children. To be included in the current study, G2 participants needed to have provided data at 17–18 years on relationships with G1 parents and peers or at 1 year postpartum on their own mental health symptoms. The resulting sample size was 270 G2 fathers with 409 G3 children.

ATP Generation 3 Study protocols have been approved by the Royal Children's Hospital Human Research Ethics Committee. Prior ATP wave study protocols were variously approved by human research ethics committees at the University of Melbourne, the Australian Institute of Family Studies and/or the Royal Children's Hospital, Melbourne.

### Measures

#### Mental Health Outcomes

G2 participants completed the short-form Depression Anxiety and Stress Scale (DASS-21) (Lovibond and Lovibond, 1995; Antony et al., 1998) at 1 year postpartum, which measured symptoms of depression (7-items, [α = 0.83], e.g., “couldn't seem to experience any positive feeling at all”), anxiety (7-items, [α = 0.71], e.g., “I was aware of dryness in my mouth”), and stress (7-items, [α = 0.84], e.g., “I found it hard to wind down”). Responses to all items were given on a 4-point scale with 1 = “not at all,” 2 = “to some degree, or some of the time,” 3 = “to a considerable degree, or a good part of the time,” and 4 = “very much, or most of the time.” Mean scores were calculated so that high scores represented increased symptoms. Given the low prevalence of elevated mental health symptoms, thresholds of ≥5, ≥4, and ≥8 were used to identify mild to severe symptoms of depression, anxiety, and stress, respectively (Lovibond and Lovibond, 1995).

#### Relationship Exposures

G2 participants completed a brief, self-reported version of the Inventory of Parent and Peer Attachment (Armsden and Greenberg, 1987) at 17–18 years, which measured degree of mutual trust (5-items parents, [α_mother_ = 0.81, α_father_ = 0.85, α_peer_ = 0.80], 4-items peers, e.g., “Respect my feelings”), quality of communication (4-items parents/peers, [α_mother_ = 0.82, α_father_ = 0.83, α_peer_ = 0.84], e.g., “I tell him/her/them about my problems and troubles”), and degree of alienation (3-items parents, [α_mother_ = 0.63, α_father_ = 0.66, α_peer_ = 0.65], 4-items peers, e.g., “I get upset more than she/he/they know(s) about”). Questions were answered about the relationships each with the participant's mother and father (step-parent if deemed as most important by the participant), and the participant's peers. Responses to all items were given on a 4-point scale with 1 = “almost always/always,” 2 = “often,” 3 = “seldom,” and 4 = “never/almost never.” Mean scores were calculated so that high scores represented higher levels on each respective dimension of trust, communication, and alienation. Validity of IPPA short forms has been demonstrated in prior studies (Laible et al., 2000, 2004; Buist et al., 2008).

#### Potential Confounding Factors

Potential confounders were selected up to the time of exposure and included G1 parent family background characteristics of country of birth (either parent not born in Australia), separation/divorce (experienced separation or divorce) and low parental education (< year 12) up until the end of G2 adolescence (ages 0–18 years). We also included G2 participant anti-social behaviour (2 behaviours at least once or 1 behaviour more frequently) across ages 13–18 years (Edwards et al., 2019), and G2 endorsement of elevated depression or anxiety symptoms prior to age 17–18 years.

### Statistical Analysis

All analyses were conducted in Stata v15 (StataCorp, 2017). Generalised estimating equations (GEEs) with an exchangeable working correlation structure (to account for within family clustering) were used to estimate a series of logistic regression analyses examining associations between adolescent relationship quality and elevated mental health at 1 year postpartum. Elevated mental health was treated as a multivariate outcome (i.e., depression, anxiety, and stress outcomes analysed simultaneously). First, elevated mental health was regressed onto each relationship quality exposure separately. Analyses were then repeated by including an interaction between relationship quality exposure and a variable denoting the type of mental health outcome (e.g., depression, anxiety, or stress) to determine if associations were similar across all outcome types. Second, elevated mental health was regressed onto all three relationship quality exposures for each relationship source (i.e., mother, father, peer). Finally, the mother and father models were repeated by including an interaction term between each parent and peer relationship quality (e.g., mother trust X peer trust; father communication X peer communication). All models were adjusted for potential confounding factors.

Multiple imputation was used to handle missing data in the inferential analyses. Twenty complete datasets were imputed, based on a multivariate normal model (Lee and Carlin, 2010). Binary variables were imputed as continuous variables and then back transformed with adaptive rounding following imputation (Bernaards et al., 2007). Estimates were obtained by pooling results across the 20 imputed datasets using Rubin's rules (Rubin, 1987).

## Results

To assess bias due to attrition, we compared G2 male participants screened for G3 study eligibility to all G2 male participants on baseline characteristics (G1 education, G1 country of birth, G2 difficult temperament, and G2 behaviour problems) measured at commencement of the ATP in 1983 (G2 age 4–8 months old). We found evidence for some selective loss of participants whose G1 parents were not born in Australia and had lower levels of education, specifically with highest achievement being high school completion or below. Those who participated in the G3 study were broadly representative of those eligible to participate on baseline characteristics.

### Descriptives

Descriptives for all analytic variables are presented in Table 1.

**Table 1:** Descriptive statistics for outcome, exposure, and potential confounding factors in the unimputed data (*n* = 270 men with 409 children).

	***n* (cases)**	**%**	**95% CI**	**% missing**
**Mental Health Symptoms**^**a**^				
Depression	26	9%	(6, 12%)	27%
Anxiety	16	5%	(3, 9%)	27%
Stress	20	7%	(4, 10%)	29%
	**m**	**SD**	**95% CI**	**% missing**
**Mother Relationships**^**b**^				
Trust	3.41	0.47	(3.35, 3.47)	19%
Communication	2.85	0.61	(2.77, 2.93)	19%
Alienation	1.98	0.58	(1.90, 2.06)	20%
**Father Relationships**^**b**^				
Trust	3.27	0.53	(3.20, 3.34)	19%
Communication	2.42	0.64	(2.34, 2.51)	19%
Alienation	2.12	0.60	(2.04, 2.20)	21%
**Peer Relationships**^**b**^				
Trust	2.98	0.30	(2.94, 3.02)	18%
Communication	2.61	0.61	(2.53, 2.69)	18%
Alienation	1.75	0.46	(1.69, 1.81)	18%
	***n*** **(cases)**	**%**	**95% CI**	**% missing**
**Potential confounding factors**^**b**^				
G1 separation	47	18%	(14, 23%)	2%
G1 non-Australian country of birth	77	30%	(25, 36%)	4%
G1 low education	53	20%	(15, 25%)	0%
G2 mental health problems	48	20%	(15, 25%)	11%
G2 delinquency	138	55%	(49, 62%)	8%

a *Assessed in G2 participants at 1-year postpartum for each G3 child,*

b *Assessed in G2 participants at 17–18 years*

### Postpartum Mental Health and Associations With Parent and Peer Relationships

Results from the GEE analyses, in which elevated 1 year postpartum mental health problems (depression, anxiety, and stress) were regressed onto late adolescent relationship exposures, are presented in Table 2. After adjustment for potential confounding factors, the strongest associations were observed for father communication and both mother and peer trust (OR range 0.60–0.62). There was no evidence that relationship quality associations differed across mental health outcome type (depression, anxiety, and stress).

**Table 2:** Regression models predicting elevated mental health symptoms (depression, anxiety, and stress).

	**Separate models**			**Simultaneously adjusted models**		
	**OR**	**95% CI**	***p***	**OR**	**95% CI**	***p***
**G1 Mother**						
Trust	0.60	(0.44, 0.82)	0.002	0.55	(0.38, 0.79)	0.001
Communication	0.72	(0.49, 1.06)	0.095	0.89	(0.56, 1.39)	0.596
Alienation	1.12	(0.75, 1.66)	0.585	0.73	(0.47, 1.15)	0.172
**G1 Father**						
Trust	0.72	(0.49, 1.05)	0.091	0.84	(0.53, 1.34)	0.465
Communication	0.60	(0.41, 0.88)	0.010	0.62	(0.40, 0.98)	0.039
Alienation	1.22	(0.80, 1.87)	0.358	0.88	(0.55, 1.43)	0.614
**Peer**						
Trust	0.62	(0.44, 0.88)	0.008	0.66	(0.44, 0.99)	0.044
Communication	0.71	(0.50, 1.02)	0.061	0.87	(0.59, 1.27)	0.464
Alienation	1.25	(0.84, 1.85)	0.272	1.00	(0.66, 1.53)	0.994

*Separate models (k = 9) assess associations between attachment construct for each source relationship individually. Simultaneously Adjusted models (k = 3) include all 3 attachment constructs for each source relationship in each regression. Both models adjusted for G1 country of birth, separation/divorce, and education; G2 anti-social behaviour, and elevated depression or anxiety symptoms during adolescence*.

Each relationship type (with mother, father, peer) was then examined by simultaneously adjusting for all relationship qualities. To help quantify these associations, the estimated prevalence of elevated symptoms of mental health problems are presented at low (−1 SD) and high (+1 SD) levels of relationship quality. For G2 relationships with G1 mothers, increases in trust (OR = 0.55; 95% CI = 0.38, 0.79) were associated with a decrease in the odds of elevated mental health problems (low trust = 10%, high trust = 3%). For G2 relationships with G1 fathers, increases in communication (OR = 0.62; 95% CI = 0.40, 0.98) were associated with a decrease in the odds of elevated mental health problems (low communication = 9%, high communication = 4%). For relationships with peers, increases in trust (OR = 0.66; 95% CI = 0.44, 0.99) were associated with a decrease in the odds of elevated mental health problems (low trust = 9%, high trust = 4%).

**Figure 1:**
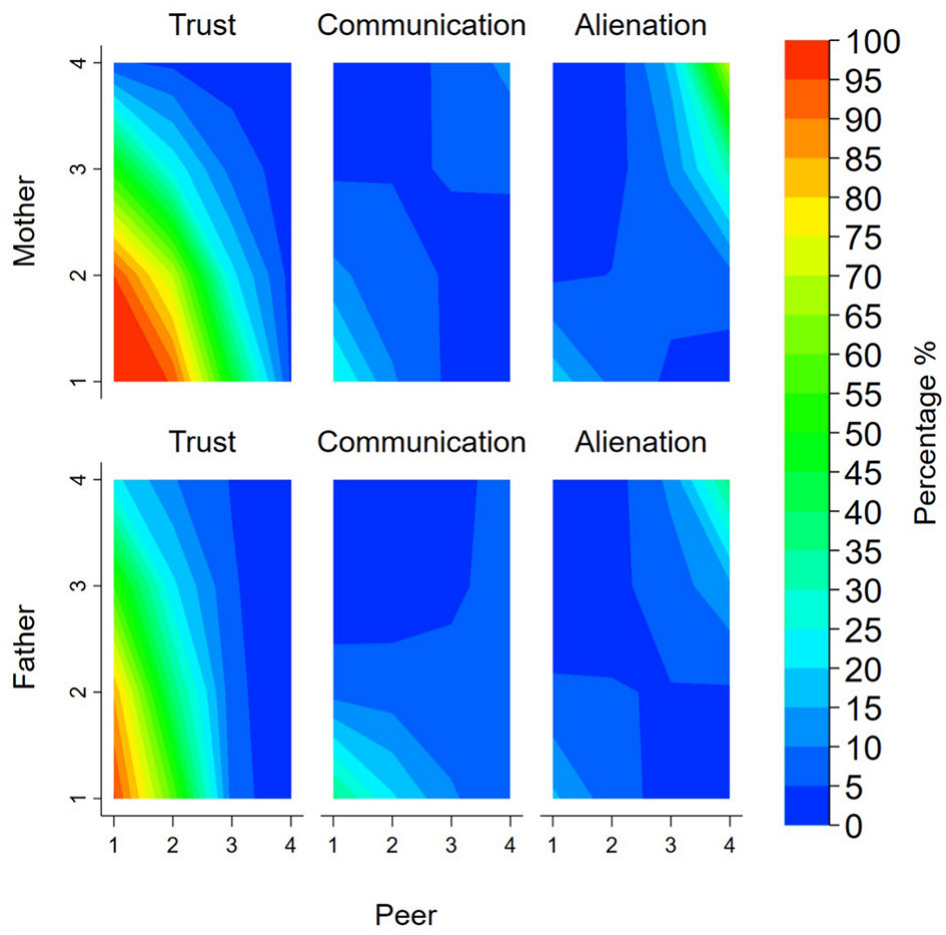
Contour plots presenting the percentage of people with elevated symptoms of mental health problems for the interaction between parent and peer relationships. Note: Colours represented percentage of people with elevated symptoms of mental health problems (blue = low percentage; green = middle percentage; red = high percentage). Peer and parent relationships presented as unstandardised scores (range 1–4).

The analyses did not provide evidence for interactions between parent (mother or father) and peer trust (mother *p* = 0.344, father *p* = 0.453), and evidence of interactions was weak for both communication (mother *p* = 0.138, father *p* = 0.057), and alienation (mother *p* = 0.078, father *p* = 0.146). Figure 1 is a visual representation of the interactions presenting estimated percentages of people with elevated symptoms of mental health problems by levels of each of the parent and peer relationship factors. In these analyses, three patterns were apparent. The most prominent was the presence of elevated symptoms of mental health problems when levels of trust, communication, and alienation were problematic in relationships with both parents and peers. The second contrasting pattern was apparent for communication and alienation whereby having extremely positive relationships with both parents and peers, was associated with elevated symptoms of mental health problems. The third pattern, also for communication and alienation, suggests that the prevalence of mental health problems was lowest when levels were discrepant between parent and peers (i.e., high for parents and low for peers or vice versa).

## Discussion

Findings from this study address a key gap in knowledge about preconception determinants of father postpartum mental health problems. We found that characteristics of adolescent male relationships with mothers, fathers, and peers were associated with subsequent risk for mental health problems once young men became fathers. Specifically, trusting adolescent relationships with mothers and peers, and good communication with fathers, were all associated with reduced risk for mental health problems in early fatherhood, more than a decade later. The associations were similar across depression, anxiety, and stress outcomes. There was additionally some weak evidence of interactions between parent and peer communication and alienation. The prevalence of elevated mental health symptoms was highest when relationships were poor with both parents and peers suggesting possible compounding effects of risk, but lowest when the relationship was poor with either parent or peer but positive with the other, suggesting possible compensatory effects.

Trust is a core feature of secure attachments across the life course and a particularly key factor in an emerging “self-concept,” or sense of identity, across the transition from adolescence to adulthood (Smetana, 2010; O'Connor et al., 2011). We found that in the mother-son relationship, diminished trust during adolescence was the most important of the three IPPA subscales in predicting later mental health problems during early fatherhood. In our fully adjusted analyses, 10% of males who experienced low levels of maternal trust in late adolescence were at risk for subsequent postpartum mental health problems, compared to 4% of males who experienced high maternal trust. In the attachment framework, a pattern of trust in early caregiving relationships creates an internalised “secure base” from which the dependent child attains confidence to explore and develop, particularly under challenging circumstances (Cassidy, 2008).

The IPPA trust scale incorporates questions about mutual feelings of acceptance, respect, and trust in judgement. Developmentally, as with secure attachment classifications, these indicators may signal the experience of parental support for autonomy in late adolescence, and a confidence in the young person's readiness to transition to adulthood (Allen and Miga, 2010; Soenens and Vansteenkiste, 2010). Prior research has demonstrated the protective nature of parent trust for adolescent mental health (Gorrese, 2016). We extend on that literature by demonstrating that trust specifically in the mother-son relationship may forge foundations of good mental health that remains apparent for more than a decade. Most importantly this protective relationship exists into early fatherhood, when it may buffer the next generation against risks that emerge from paternal mental health problems.

It is possible that because mothers are typically primary caregivers, the experience of trust within the adolescent mother-son relationship may be of particular importance once men become caregivers of their own children. This may be intensified in the context of contemporary attitudes and beliefs about fatherhood in which many men report a desire to be nurturant, emotionally involved caregivers, in a manner more resembling the care they experienced from their mothers than fathers (Kings et al., 2017). Across the transition from care receiver to caregiver, trust in the relationship with one's own primary caregiver has the capacity to foster self-regulation and care for others (Scharf and Goldner, 2018). Aligned to this is prospective research with females that shows that during adolescence, the experience of maternal but not paternal psychological control—a construct aligned to low trust—is associated with impairments in the emotional bonds formed with next generation infants in the first year postpartum (Macdonald et al., 2018). The current study, along with the prior research, combine to suggest that regardless of gender, a history of trust with one's mother, or possibly the primary attachment figure during adolescence has implications for later emotional functioning in the postpartum period of parenthood.

Furthermore, we found that for peer relationships in adolescence, trust was also the most important of the three IPPA factors in predicting later mental health problems during early fatherhood. Adolescent males who reported high, compared to low, trust in their peer relationships had substantially reduced risk of postpartum mental health problems. In fully adjusted analyses, 9% of those who experienced low trust in peers during adolescence were at subsequent risk for postpartum mental health problems, compared to 4% for those with high peer trust. These findings align with meta-analysed results (k = 7) supporting a risk association between peer trust and depression (−0.22, *p* < 0.001) reported by Gorrese (2016). However, outcomes in that meta-analysis were restricted to adolescence. Our results show that effects persist over much longer periods of time, into the postpartum period. Meta-analyses have also found that adolescent males report lower trust in relationships with peers than females (Gorrese and Ruggieri, 2012). This suggests that not only are interventions on trust in relationships that target adolescent males particularly important, they may also have intergenerational benefits. That trust in relationships with both mothers and peers were predictive of men's postpartum mental health may indicate generalisation of working models of trust from the primary attachment figure to peers. This possibility aligns with attachment theory and supports the proposition that secure representations from both original and subsequent attachment relationships are important in promoting effective emotion regulation (Bowlby, 1973, 1979; Furman et al., 2002).

The pattern of results we observed for relationships that adolescent boys had with their fathers was different. Here we found that communication was the more important of the three IPPA subscales. When quality of father communication was low, the rate of paternal postpartum mental health risk was 9.5%, compared to 4% when communication was high. The communication subscale indicates the degree of encouragement of and mutuality in sharing of opinions, and the nature of the parents' intuitive communication, especially in challenging affective contexts. As such, high endorsement of these items is a proxy for the degree to which the father was experienced as a “safe haven” for the boy during adolescent development (Crowell et al., 2008). Low levels of personal disclosure between fathers and sons are characteristic of socialised emotional distancing that is a feature of dominant forms of masculinity (Emslie et al., 2006). Rigid adherence by men to restrictive masculine values has been linked to elevated risk for mental health problems (Gerdes and Levant, 2017). Similarly, in the parenting literature, emotion-dismissing behaviours that cut-off a child's communication about problems also heightens risk of poor social and emotional outcomes (Kehoe et al., 2014). Our results appear to indicate multi-generational effects of such emotional distancing. Here, when Generation 1 fathers were reported to be unaware of their sons' troubles or could not be depended upon to hear out a son's difficulties, there was substantially increased risk for postpartum mental health problems.

We also assessed interactions between mothers and peers, and fathers and peers, for each of the factors of trust, communication, and alienation. This allowed us to assess whether positive qualities of the relationship with one might compensate for negative qualities with the other and whether negative relationships with both compounded risk for subsequent paternal mental health problems. In light of our low-powered environment (small sample), there was weak evidence to support an interaction between parent and peer levels of communication and alienation, suggesting patterns of a compensatory effect. Surprisingly, however, symptoms of mental health problems were least prevalent when parent and peer levels were discrepant; whereas, when levels were positive for both parent and peer relationships, an increase in the prevalence of mental health problems was observed. As noted above, inferences about interactions should be interpreted with caution because the available sample precluded well-powered evaluations. However, if replicated, strengthening of alternative relationships when key attachment supports fail may play a role in preventing postnatal distress in men, in line with the proposition that at least a single source of attachment security is essential (Bowlby, 1973).

Additionally, a possible explanation for the comparatively higher level of postpartum mental health risk observed among those with extreme positive levels (high communication or low alienation) from both parents and peers may be indicative of effects of idealised rather than realistic appraisals of relationships. Prior studies suggest a normative curvilinear trajectory of adolescent to young adult self-reported relationship quality with parents. This is characterised by a decline in reported relationship quality typical in late adolescence, presenting as a transient dip between reports of high quality relationships in early adolescence and then again in young adulthood (Wintre et al., 1995; Koepke and Denissen, 2012). This trajectory arguably supports the developmental process of autonomy and individuation (Koepke and Denissen, 2012). Similarly, while adolescence is a period of strengthening relationships with peers, some adolescents report being enmeshed in peer relationships indicating immaturity and a lack of emotional independence (Mayseless and Scharf, 2009). While speculative, it is possible that in our study extreme positive self-reports of relationships in late adolescence may indicate a level of dependency and failure or delay to progress in normative developmental processes (Koepke and Denissen, 2012). This potential mechanism in the development of some fathers' postpartum mental health problems is worthy of future investigation using specific measures of dependence or enmeshment and a multi-informant design.

## Implications

Relationship functioning is modifiable (Fonagy et al., 2015). Past research supports the implementation of prevention programs that target relationships with parents and peers to improve proximal mental health outcomes for adolescents (Catalano et al., 2004; Kehoe et al., 2014; Rose et al., 2014). Our research provides early evidence to support the possibility that such programs may produce mental health benefits years later into the next generation of family life. Existing universal and targeted services and programs delivered in school and community settings rarely focus on parent and peer attachment (Werner-Seidler et al., 2017). However, many of those based on the cognitive-behavioural model include components which may improve interpersonal relationships (e.g., social skills; conflict resolution) (Dray et al., 2017). Furthermore, there are some promising results from programs which include a focus on building healthy attachments (Rose et al., 2014); these may be especially impactful if they incorporate parent involvement (Catalano et al., 2004). Community-based parenting programs designed to teach parents skills in responding to emotions and foster closer parent-adolescent connection have also demonstrated positive effects for both parents and adolescents (Havighurst et al., 2013, 2019; Toumbourou et al., 2013; Kehoe et al., 2014). Additionally, attachment-based treatments for adolescents are increasing and could help to inform universal programs (Kobak et al., 2015).

Prevention targets for men are of particular importance in a context where formal perinatal supports within health services are focused predominantly on mothers and infants (Panter-Brick et al., 2014; Allport et al., 2019; Hodgson et al., 2021). Of note are growing calls for gender-specific programs for males during adolescence when a divergence in mental health outcomes, gender-based identities, behavioural risks, and barriers to support, start to become pronounced (Patton et al., 2018; Rice et al., 2018). Our research suggests that adolescent boys might benefit differentially from different components of relationships (i.e., trust in peers and mothers and communication with fathers). Additionally, given evidence that male mental health problems, including paternal depression, often present with increased levels of anger (Macdonald et al., 2020), a focus on male-specific preventative targets may also have flow on effects for partners and children if they improve family harmony and reduce conflict.

## Strengths and Limitations

A strength of this study is the focus on pathways to paternal mental health. The imbalance in literature favouring investigation of psychological functioning of mothers is slowly being addressed; however, we present here one of the few studies that has prospectively addressed factors that might be appropriate targets for prevention of paternal postpartum mental health problems. Our longitudinal study design allowed us to identify characteristics of relationships that potentially shape the futures of adolescent boys into adulthood and fatherhood. However, limitations of longitudinal studies such as ours include missing data and biases that may be associated with non-responses. Within the achieved sample, levels of missing data were low and addressed using multiple imputation. Nonetheless as with all longitudinal studies, some bias due to differential attrition is likely. While the participating G2s in our sample were broadly similar to those eligible on baseline characteristics, compared to the original sample, families retained in the study were more often born in Australia and had higher education levels. Future research could investigate these associations in specific population groups.

Our study may also be limited by the use of self-reports, which are subject to social desirability biases (van de Mortel, 2008) and mood states (Fergusson et al., 1993; Robinson and Clore, 2002). While inclusion of observational measures and other informants is warranted in future research, our findings nonetheless align with prior research demonstrating the value and substantial predictive utility of adolescents' own perceptions of their parental and peer relationships (Chen et al., 2019a,b). Future research would also benefit from investigating associations between preconception parent and peer relationships and later clinical diagnoses of mental health problems, although non-clinical levels of depression and anxiety are associated with poor psychosocial functioning (Gotlib et al., 1995; Letcher et al., 2012).

We adjusted for key demographic variables and prior levels of the outcome, which can account not only for reverse causation but also confounding by other common causes of exposure and outcome (VanderWeele, 2013). As with all observational studies, though, potential for unmeasured confounding remains. Future studies should explore the potential causal nature of these associations using alternative designs (Thapar and Rutter, 2019). There are, additionally, proximal factors that may be relevant to paternal mental health include parity, multiple births, and perinatal outcomes such as gestational age and infant health. We did not include these in our analyses because they are potentially mediating mechanisms on the causal pathways of the associations we tested, and their inclusion may have concealed total effects. Nevertheless, future research, with larger samples, could explore their contribution to associations between preconception relationships and paternal mental health outcomes.

## Conclusion

Supporting the positive development of boys into fatherhood requires careful investment in the quality of their attachment relationships with mothers, fathers, and peers over time. Prior research has established the importance of trust and communication in parent and peer relationships during adolescence (Gorrese and Ruggieri, 2012; Gorrese, 2016). Here, we provide evidence suggesting enduring effects, such that boys who experienced trust with their mothers and peers and positive communication with their fathers were at substantially reduced risk of postpartum mental health problems more than a decade later. Our finding that different factors in relationships with mothers and fathers were differentially related to mental health outcomes may be particularly relevant as momentum grows for gender-specific programs that support the social and emotional development of adolescent males (Patton et al., 2018; Rice et al., 2018). Supporting young males to resolve issues that may impede development of trust in peer relationships may also have far-reaching benefits into fatherhood. Relationships that build trust, and that are based on good communication, may not only strengthen boys' social and emotional development in their adolescent years, they may also build the foundations for better mental health that, our findings suggest, endure into fatherhood.

## Data Availability Statement

The datasets presented in this article are not readily available because ethics approvals do not permit the data to be made publicly available due to limitations of participant consent and concerns regarding potential re-identifiability. Requests to access the datasets should be directed to https://lifecourse.melbournechildrens.com/data-access/ where submissions can be made using our institutional data access protocols.

## Ethics Statement

Ethics approvals for data collection in the multiple waves of the ATP and its Generation 3 study were variously granted by the human research ethics committees of the Royal Children's Hospital in Melbourne, the University of Melbourne, and/or the Australian Institute of Family Studies. ATPG3 participants provided written informed consent to participate in the study.

## Author Contributions

JAM, CO, CG, PL, and ES: conceptualisation and study design. CG: statistical analyses. CO, PL, AS, JAM, CG, DH, JT, BE, and JEM: ATP cohort experts. JAM, PL, ES, KM, JEM, KT, CD, EB, and JC: literature review. JAM, CG, CO, PL, ES, and KT: interpretation of results. JAM, PL, ES, and CG: drafting of original introduction and conclusion, and drafting of original methods and results. All authors: critical revision of manuscript and final acceptance of manuscript.

## Conflict of Interest

The authors declare that the research was conducted in the absence of any commercial or financial relationships that could be construed as a potential conflict of interest.
